# Facile synthesis of TiO_2_/Ag_3_PO_4_ composites with co-exposed high-energy facets for efficient photodegradation of rhodamine B solution under visible light irradiation

**DOI:** 10.1039/d0ra04183a

**Published:** 2020-06-26

**Authors:** Yi-en Du, Wanxi Li, Yang Bai, Zewen Huangfu, Weijin Wang, Ruidong Chai, Changdong Chen, Xiaojing Yang, Qi Feng

**Affiliations:** School of Chemistry & Chemical Engineering, Jinzhong University Jinzhong 030619 China duyien124@163.com; College of Chemistry, Chemical Engineering and Environmental Engineering, Liaoning Shihua University Fushun 113001 China chencd1984@gmail.com; Beijing Key Laboratory of Energy Conversion and Storage Materials, College of Chemistry, Beijing Normal University Beijing 100875 China yang.xiaojing@bnu.edu.cn; Department of Advanced Materials Science, Faculty of Engineering, Kagawa University 2217-20 Hayashi-cho Takamatsu-shi 761-0396 Japan

## Abstract

In this study, TiO_2_/Ag_3_PO_4_ composites based on anatase TiO_2_ nanocrystals with co-exposed {101}, {010}/{100}, {001} and [111]-facets and Ag_3_PO_4_ microcrystals with irregular and cubic-like polyhedron morphologies were successfully synthesized by combining hydrothermal and ion-exchange methods. The anatase TiO_2_ nanocrystals with different high-energy facets were controllably prepared *via* hydrothermal treatment of the exfoliated [Ti_4_O_9_]^2−^/[Ti_2_O_5_]^2−^ nanosheet solutions at desired pH values. The Ag_3_PO_4_ microcrystal with different morphologies was prepared *via* the ion-exchange method in the presence of AgNO_3_ and NH_4_H_2_PO_4_ at room temperature, which was used as a substrate to load the as-prepared anatase TiO_2_ nanocrystals on its surface and to form TiO_2_/Ag_3_PO_4_ heterostructures. The apparent rate constant of the pH 3.5-TiO_2_/Ag_3_PO_4_ composite was the highest at 12.0 × 10^−3^ min^−1^, which was approximately 1.1, 1.2, 1.4, 1.6, 13.3, and 24.0 fold higher than that of pH 0.5-TiO_2_/Ag_3_PO_4_ (10.5 × 10^−3^ min^−1^), pH 7.5-TiO_2_/Ag_3_PO_4_ (10.2 × 10^−3^ min^−1^), pH 11.5-TiO_2_ (8.8 × 10^−3^ min^−1^), Ag_3_PO_4_ (7.7 × 10^−3^ min^−1^), blank sample (0.9 × 10^−3^ min^−1^), and the commercial TiO_2_ (0.5 × 10^−3^ min^−1^), respectively. The pH 3.5-TiO_2_/Ag_3_PO_4_ composite exhibited the highest visible-light photocatalytic activity which can be attributed to the synergistic effects of its heterostructure, relatively small crystal size, large specific surface area, good crystallinity, and co-exposed high-energy {001} and [111]-facets. The as-prepared TiO_2_/Ag_3_PO_4_ composites still exhibited good photocatalytic activity after three successive experimental runs, indicating that they had remarkable stability. This study provides a new way for the preparation of TiO_2_/Ag_3_PO_4_ composite semiconductor photocatalysts with high energy crystal surfaces and high photocatalytic activity.

## Abstract 2

TiO_2_/Ag_3_PO_4_ composites with co-exposed {101}, {010}/{100}, {001} and [111]-facets were successfully synthesized by combining hydrothermal and ion-exchange methods.

## Graphical abstract

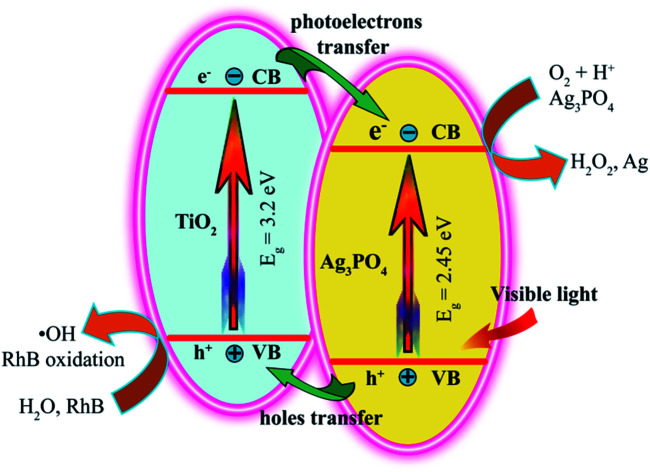

## 1. Introduction

With the rapid development of industrialization, energy and environmental crises have become the key factors restricting the sustainable development of human society. Therefore, it is very urgent to search for suitable semiconductor photocatalysts to make full use of solar energy to split water into hydrogen, convert carbon dioxide into fuels, store energy, and degrade the organic wastewater discharged from the textile industry.^1–5^ In recent decades, different types of semiconductor photocatalysts, such as carbon-cloth functionalized transition metal based electrocatalysts,^6^ quantum dot-based photocatalysts,^7–9^ iron (Fe)-doped ZrO_2_,^10^ metal–organic framework (MOF)-based heterostructured catalysts,^11^ and ZnO/Bi_2_WO_6_ nanohybrids^12^ have been reported. Among the well-known oxide semiconductor photocatalysts, titanium dioxide (TiO_2_) has been proven to be the best choice due not only to its excellent photo-oxidization ability and low cost but also its long-term photostability and chemical stability and innocuousness.^13,14^ However, the photocatalytic efficiency of TiO_2_ still needs to be further improved for its practical application. The photocatalytic efficiency of TiO_2_ is mainly dependent on the phase structure, crystallization, crystal size, specific surface area, and surface energy.^15,16^ However, based on the principle of surface energy minimization (0.44 J m^−2^ for {101} facets < 0.53 J m^−2^ for {010}/{100} facets < 0.90 J m^−2^ for {001} facets < 1.09 J m^−2^ for {110} facets < 1.61 J m^−2^ for {111} facets), the proportion of high-energy crystal surfaces in the natural and synthetic anatase TiO_2_ crystals under equilibrium condition is very small, resulting in the dominant exposed {101} crystal facets (more than 94%) on its surface.^17,18^ Since the pioneering work by Wen and co-workers on the synthesis of nanometer-sized anatase TiO_2_ crystals with a large percentage of {101} facets by using the delaminated [Ti_1.73_O_4_]^1.07−^ nanosheets as the precursor, there has been intensive interest in the flexible and controllable synthesis of anatase crystals with varied high-energy facets, such as {001}, {010}/{100}, {110} and {111} facets.^19–22^ Recently, we synthesized high-energy {010}, {001}, and [111]-faceted anatase TiO_2_ nanocrystals by using the delaminated [Ti_4_O_9_]^2−^ and [TiO_3_]^2−^ nanosheets as the precursors in the presence and absence of capping agent, which displayed superior photocatalytic and photovoltaic performance.^23–25^ Although the exposed high-energy crystal surface of anatase TiO_2_ crystals will be conducive to improving the photocatalytic activity and dye-sensitized solar energy performance, however, anatase TiO_2_ crystals cannot suitable for applications under visible light irradiation due to its wide band gap (3.2 eV), resulting in the lower energy conversion efficiency in practical application.^26^

In order to overcome above limitation, it is of great significance to extend the light absorption range of the anatase TiO_2_ crystals to the visible light region.^27–29^ Silver orthophosphate (Ag_3_PO_4_) is a semiconductor photocatalyst with a narrow band gap of 2.45 eV, and is often to decompose organic contaminants and oxidize water to produce oxygen under visible light irradiation.^30^ However, the narrow band gap energy and low valence band (VB) and conduction band (CB) position of Ag_3_PO_4_ result in high recombination rate and weak redox capacity of photogenerated electrons and holes, which severely weaken the photocatalytic activity of Ag_3_PO_4_.^26,31^ Therefore, it is an effective strategy to form a heterojunction by coupling anatase TiO_2_ crystals with Ag_3_PO_4_ photocatalyst for improving the photocatalytic activity under visible-light irradiation.^32,33^ Zhang *et al.* synthesized one-dimensional heterostructured Ag_3_PO_4_/TiO_2_ photocatalyst with improved photocatalytic activity for degradation of 4-nitrophenol in simulant wastewater under visible light.^34^ An *et al.* reported that the floating HGMs-TiO_2_/Ag_3_PO_4_ composites exhibited superior photocatalytic performance than that of pure Ag_3_PO_4_ and TiO_2_/Ag_3_PO_4_ for degradation of methylene blue solution under visible light irradiation.^3^ Xu *et al.* reported that the magnetic Ag_3_PO_4_/TiO_2_/Fe_3_O_4_ heterostructured nanocomposite showed enhanced photocatalytic performance for the degradation of acid orange 7 under visible light irradiation.^35^ Hamrouni *et al.* synthesized Ag doped TiO_2_–Ag_3_PO_4_ (Ag@TiO_2_–Ag_3_PO_4_) composites by coupling sol–gel and precipitation methods, which significantly improved the photocatalytic activity than that of the TiO_2_–Ag_3_PO_4_ and the benchmark TiO_2_ Evonik P25 for degradation of 4-nitrophenol solution under solar light irradiation.^36^

In this study, anatase TiO_2_ nanocrystals with different high energy facets were successful synthesized by using the exfoliated two-dimensional [Ti_4_O_9_]^2−^/[Ti_2_O_5_]^2−^ nanosheets, which were compounded with Ag_3_PO_4_ microcrystals to form a series of heterostructured TiO_2_/Ag_3_PO_4_ composites. To our knowledge, this is the first time to study the TiO_2_/Ag_3_PO_4_ photocatalysts formed by the combination of the anatase TiO_2_ nanocrystals with high energy crystal surface and Ag_3_PO_4_ with different morphologies. Various catalyst characterization of the synthesized TiO_2_/Ag_3_PO_4_ composites confirmed that TiO_2_ nanocrystals with co-exposed high-energy facets were successfully attached to the surface of Ag_3_PO_4_ microcrystals. In comparison to the commercial TiO_2_ and the pure Ag_3_PO_4_ samples, the heterostructured TiO_2_/Ag_3_PO_4_ composites exhibited good photocatalytic activity for the degradation of rhodamine B under visible light irradiation, which can be attributed to the separation of the e^−^ (in Ag_3_PO_4_ crystal) and h^+^ (in TiO_2_ nanocrystal) inhibits the charge recombination. For the as-prepared TiO_2_/Ag_3_PO_4_ composites, the pH 3.5-TiO_2_/Ag_3_PO_4_ exhibited the highest photocatalytic activity, which can be attributed to the synergistic effects of its relative small crystal size, large specific surface area, good crystallinity, and co-exposed high-energy {001} and [111]-facets. However, although the as-prepared TiO_2_/Ag_3_PO_4_ composites exhibited good stability, the photocatalytic performance needs to be further improved for their practical application.

## 2. Materials and methods

### 2.1 Synthesis of TiO_2_ nanocrystals

The K_2_Ti_4_O_9_/K_2_Ti_2_O_5_·*x*H_2_O composite was prepared *via* solid-state synthesis in the present of 14.5109 g K_2_CO_3_ (0.105 mol, Tianjin Bodi Chemical Co., Ltd., Tianjin, China) and 15.9800 g TiO_2_ (0.200 mol, Tianjin Bodi Chemical Co., Ltd., Tianjin, China) at 900 °C for 24 h. The obtained K_2_Ti_4_O_9_/K_2_Ti_2_O_5_·*x*H_2_O composite (10.0 g) was dissolved in 1 M HCl aqueous (1 L, Sichuan Xilong Chemical Co., Ltd., Chengdu, China) for three days under continuous magnetic stirring conditions to obtain H_2_Ti_4_O_9_·H_2_O/H_2_Ti_2_O_5_·H_2_O composite. Then, 6.0 g H_2_Ti_4_O_9_·H_2_O/H_2_Ti_2_O_5_·H_2_O composite, 7.0 g tetramethylammonium hydroxide (TMAOH, Shanghai Dibai Biological Technology Co., Ltd., Shanghai, China) and 50 mL deionized water were mixed uniformly, which were hydrothermally treated at 100 °C for 24 h in a homogenous reaction (KLJX-8A, Yantai Keli Chemical Equipment Co. Ltd., Yantai, China) under stirring conditions to prepare TMA^+^-intercalated H_2_Ti_4_O_9_·H_2_O/H_2_Ti_2_O_5_·H_2_O compound. The resulting white TMA^+^-intercalated compound was dispersed in 500 mL of deionized water under stirring conditions for three days to obtain the nanosheets solutions containing of H_2_Ti_4_O_9_/H_2_Ti_2_O_5_ compound. The above nanosheets solutions were adjusted to desired pH values (0.5–11.5) at 180 °C for 24 h to prepared TiO_2_ nanocrystals.

### 2.2 Synthesis of Ag_3_PO_4_ crystals

Silver orthophosphate (Ag_3_PO_4_) crystals were synthesized by using an ion-exchange method, using AgNO_3_ (Beijing Beihua Fine Chemicals Co., Ltd., Beijing, China) and NH_4_H_2_PO_4_ (Beijing Guangfu Technology Development Co., Ltd., Beijing, China) as the starting materials and NH_4_F (Tianjin Bodi Chemical Co., Ltd., Tianjin, China) as a capping agent at room temperature. The Ag_3_PO_4_ sample was prepared according to the stoichiometry of AgNO_3_ and NH_4_H_2_PO_4_ to 3 : 1 (3AgNO_3_ + NH_4_H_2_PO_4_ = Ag_3_PO_4_↓ + NH_4_NO_3_ + 2HNO_3_). In a typical synthesis, 3.8228 g (0.0225 mol) AgNO_3_ and 0.8626 g (0.0075 mol) NH_4_H_2_PO_4_ were dissolved in 150 mL of deionized water, respectively. Then, 1.0 g NH_4_F (0.027 mol) was dissolved in the NH_4_H_2_PO_4_ aqueous solution. After that, the AgNO_3_ solution (0.15 mol L^−1^) was transferred to pear-shaped separatory funnel and added to the NH_4_H_2_PO_4_ aqueous solution (0.05 mol L^−1^) drop by drop under continuous magnetic stirring. The yellow Ag_3_PO_4_ precipitate was obtained after 2 h, which was washed with lots of deionized water to remove unwanted ions, kept in the dark and dried at ambient temperatures.

### 2.3 Synthesis of TiO_2_/Ag_3_PO_4_ composites

The well dispersed TiO_2_ colloidal suspensions were obtained by dispersed 1.2 g as-prepared TiO_2_ in 200 mL deionized water under stirring for 2 h. Then, 0.3 g Ag_3_PO_4_ precipitate was added to the above TiO_2_ colloidal suspensions and kept under stirring for 2 h to generate TiO_2_/Ag_3_PO_4_ composites (*w*(TiO_2_) = 80%, *w*(Ag_3_PO_4_) = 20%). Finally, the composites were collected by filtering, which were washed several times, and dried at room temperature.

### 2.4 Characterization

The crystal structure of obtained samples were characterized by powder X-ray diffractometer (XRD) on a XRD-6100 (Shimadzu, Kyoto, Japan) with monochromated Cu Kα radiation (*λ* = 1.5406 Å). The data were collected for scatting angles (2*θ*) from 5 to 80° with a scanning speed of 8° min^−1^. The morphology of the samples were investigated by using cold field emission scanning electron microscope (FESEM, JSM-7500F, Japan). The crystalline nanostructures were investigated using transmission electron microscopy (TEM), high-resolution transmission electron microscopy (HRTEM) (Tecnai G^2^ F20 S-TWIN, FEI, America). The specific surface areas of the as-prepared samples were determined by using the Brunauer–Emmett–Teller (BET) method (Autosorb-IQ3, Quantachrome, America). UV-Vis-NIR spectra of the samples were obtained by using a Cary Series UV-Vis-NIR Spectrophotometer (Agilent Technologies, Cary 5000). The absorbance of rhodamine B solution was recorded within the wavelength range of 350–650 nm by using a TU-1901 UV-vis spectrophotometer (Beijing Purkinje General Instrument Co. Ltd).

### 2.5 Photocatalytic activity evaluations

The photocatalytic activities of the as-synthesized TiO_2_/Ag_3_PO_4_ composites were evaluated by monitoring the degradation of rhodamine B (RhB). The irradiation source was provided by a 300 W xenon lamp equipped with a 400 nm cutoff light filter and the wavelength ranges from 400 nm to 600 nm. Typically, 75 mg TiO_2_/Ag_3_PO_4_ composite was suspended in 150 mL RhB solution (10 ppm). Prior to illumination, the suspensions were magnetically stirred for 2 h in the dark to achieve adsorption–desorption equilibrium. At intervals of 15 min, 5 mL of suspensions were taken out and centrifuged at 2500 rpm for 10 min to remove the TiO_2_/Ag_3_PO_4_ composites. The changes of RhB concentration during xenon light irradiation were determined by using a TU-1901 ultraviolet-visible spectrophotometer at the maximum absorption wavelength of RhB (554 nm) with deionized water as the reference solution. For comparison, the commercial TiO_2_ powder (∼70.9% anatase and ∼29.1% rutile), and as-prepared Ag_3_PO_4_ powder were also used as the photocatalytic references. The stability and recyclability of the TiO_2_/Ag_3_PO_4_ composites were investigated by the degradation experiments of the 10 ppm RhB solution (150 mL).

## 3. Results

### 3.1 Structure and morphological characterization

**Fig. 1:**
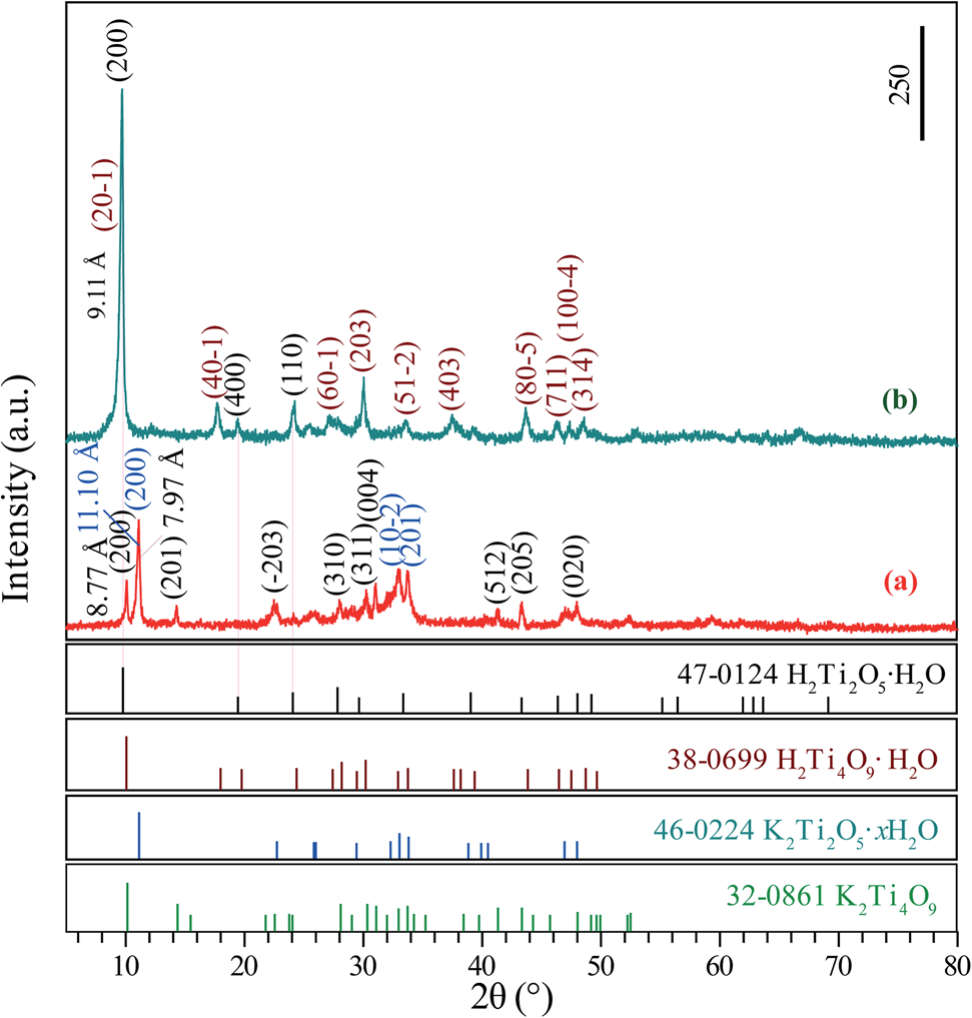
XRD patterns of the layered (a) K_2_Ti_4_O_9_/K_2_Ti_2_O_5_·*x*H_2_O composite and the protonic titanate (b) H_2_Ti_4_O_9_·H_2_O/H_2_Ti_2_O_5_·H_2_O composite.

The XRD patterns of the K_2_Ti_4_O_9_/K_2_Ti_2_O_5_·*x*H_2_O and H_2_Ti_4_O_9_·H_2_O/H_2_Ti_2_O_5_·H_2_O composites depicted characteristic structures. The diffraction peaks at 10.08°, 14.23°, 22.49°, 28.08°, 30.24°, 31.04°, 41.30°, 43.34° and 48.04° corresponded to the (200), (201), (−203), (310), (311), (004), (512), (205) and (020) crystal facets of K_2_Ti_4_O_9_, which depicted the characteristic monoclinic structure of K_2_Ti_4_O_9_ (JCPDS card no. 32-0861, space group: *C*2/*m*, lattice parameter: *a* = 19.968 Å, *b* = 3.746 Å, *c* = 12.025 Å and *β* = 114.01°) (Fig. 1(a)). The diffraction peaks at 11.10°, 32.96°and 33.78° corresponded to the (200), (10–2) and (201) crystal facets of K_2_Ti_2_O_5_·*x*H_2_O, which depicted the characteristic monoclinic structure of K_2_Ti_2_O_5_·*x*H_2_O (JCPDS card no. 46-0224, space group: *C*2/*m*, lattice parameter: *a* = 6.605 Å, *b* = 3.069 Å, *c* = 5.665 Å and *β* = 99.98°) (Fig. 1(a)). Fig. 1(b) shows XRD patterns of proton exchanged phase, the diffraction peaks located at 9.70°, 17.70°, 22.49°, 30.04°, 37.48° and 43.72° are ascribed to (20–1), (40–1), (203), (403) and (80–5) crystal facets of H_2_Ti_4_O_9_·H_2_O, respectively, and the diffraction peaks located at 9.70°, 19.40° and 24.22° are ascribed to (200), (400) and (110) crystal facets of H_2_Ti_2_O_5_·H_2_O, respectively. The basal spacing was changed from 8.77 Å for K_2_Ti_4_O_9_ (or 11.10 Å for K_2_Ti_2_O_5_·*x*H_2_O) to 9.11 Å for H_2_Ti_4_O_9_·H_2_O (or 9.11 Å for H_2_Ti_2_O_5_·H_2_O), indicating the protonation of K_2_Ti_4_O_9_/K_2_Ti_2_O_5_·*x*H_2_O composite occur successfully. Based on the above analysis, it can be seen that the K_2_Ti_4_O_9_/K_2_Ti_2_O_5_·*x*H_2_O composite and the protonated products H_2_Ti_4_O_9_·H_2_O/H_2_Ti_2_O_5_·H_2_O have been successfully prepared.

**Fig. 2:**
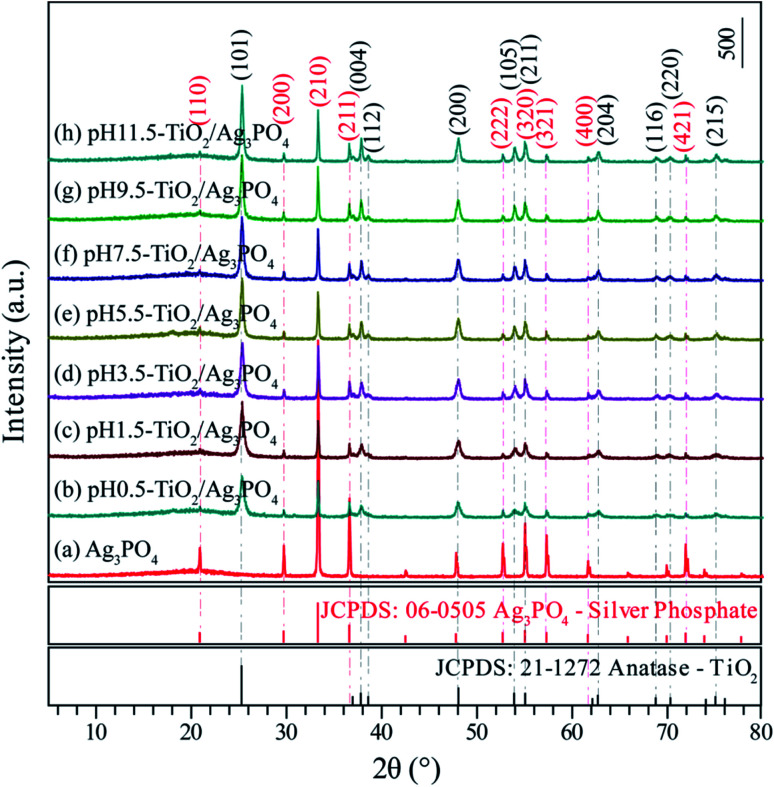
XRD patterns of the (a) Ag_3_PO_4_, (b) pH 0.5-TiO_2_/Ag_3_PO_4_, (c) pH 1.5-TiO_2_/Ag_3_PO_4_, (d) pH 3.5-TiO_2_/Ag_3_PO_4_, (e) pH 5.5-TiO_2_/Ag_3_PO_4_, (f) pH 7.5-TiO_2_/Ag_3_PO_4_, (g) pH 9.5-TiO_2_/Ag_3_PO_4_, and (h) pH 11.5-TiO_2_/Ag_3_PO_4_ composites specimens obtained from the exfoliated H_2_Ti_4_O_9_/H_2_Ti_2_O_5_·H_2_O nanosheets composites.

Fig. 2(a) presents the XRD pattern of the Ag_3_PO_4_ obtained by simple ion-exchange method. The diffraction peaks at 20.89°, 29.72°, 33.12°, 36.60°, 52.72°, 55.06°, 57.30°, 61.68° and 71.92° corresponded to the (110), (200), (210), (211), (222), (320), (321), (400) and (421) crystal facets of Ag_3_PO_4_, which depicted the characteristic cubic structure of Ag_3_PO_4_ (JCPDS card no. 06-0505, space group: *P*43̄*n*, lattice parameter: *a* = 6.013 Å and *β* = 90°) (Fig. 1(a)). After hydrothermal treatment of the exfoliated H_2_Ti_4_O_9_/H_2_Ti_2_O_5_·H_2_O nanosheets composites, both composites transformed into anatase phase TiO_2_ completely. The TiO_2_/Ag_3_PO_4_ composites were prepared by mixed the obtained anatase TiO_2_ nanocrystals and Ag_3_PO_4_ in water. Fig. 2(b–h) shows the XRD patterns of the obtained TiO_2_/Ag_3_PO_4_ composites, except for the characteristic diffraction peaks of the Ag_3_PO_4_ crystals, other diffraction peaks at around 25.32°, 37.84°, 38.60°, 48.06°, 53.98°, 55.04°, 62.84°, 68.82°, 70.34° and 75.24° corresponded to the (101), (004), (112), (200), (105), (211), (204), (116), (220) and (215) crystal facets of anatase TiO_2_ (JCPDS card no. 21-1272, crystal system: tetragonal, space group: *I*4_1_/*amd*, lattice parameter: *a* = 3.7852 Å and *c* = 9.5139 Å). The diffraction peaks of TiO_2_/Ag_3_PO_4_ composites are shifted slightly to the right, which can be attributed to the basic crystal plane spacing (*d*_basic_) of the crystal plane varies slightly. The *d*_basic_ values of TiO_2_(101) (Ag_3_PO_4_(110)) are 3.514 (4.244), 3.509 (4.236), 3.507 (4.234), 3.512 (4.247), 3.515 (4.250), 3.512 (4.244), and 3.512 Å (4.244 Å) for the pH 0.5-TiO_2_/Ag_3_PO_4_, pH 1.5-TiO_2_/Ag_3_PO_4_, pH 3.5-TiO_2_/Ag_3_PO_4_, pH 5.5-TiO_2_/Ag_3_PO_4_, pH 7.5-TiO_2_/Ag_3_PO_4_, pH 9.5-TiO_2_/Ag_3_PO_4_, and pH 11.5-TiO_2_/Ag_3_PO_4_, respectively. Moreover, for the as-prepared pH 5.5-TiO_2_/Ag_3_PO_4_ composite, a weak impurity peak was observed at 18.04°. The intensities of anatase TiO_2_ and Ag_3_PO_4_ crystals indicate that the TiO_2_/Ag_3_PO_4_ composites are well crystallized and no diffraction peaks attributed to rutile or brookite are detected. It can be seen that with increasing pH value, the peak intensities of anatase TiO_2_ increase and the width of the (101) crystal facets diffraction peak of anatase TiO_2_ (2*θ* = 25.32°) become narrow, indicating the increase of the average crystalline sizes and relative crystallinity of the TiO_2_/Ag_3_PO_4_ composites. The diffraction peaks of TiO_2_(101)/Ag_3_PO_4_ composites synthesized are relatively broad, which may be ascribed to the small size of TiO_2_/Ag_3_PO_4_ composites. Based on the broadening of (101) peaks of the TiO_2_/Ag_3_PO_4_ composites specimens (b–h) in Fig. 2, the average crystalline size of the specimens can be calculated as 23.0, 23.2, 23.5, 26.4, 28.0, 29.7, and 32.0 nm for pH 0.5-TiO_2_/Ag_3_PO_4_, pH 1.5-TiO_2_/Ag_3_PO_4_, pH 3.5-TiO_2_/Ag_3_PO_4_, pH 5.5-TiO_2_/Ag_3_PO_4_, pH 7.5-TiO_2_/Ag_3_PO_4_, pH 9.5-TiO_2_/Ag_3_PO_4_, pH 11.5-TiO_2_/Ag_3_PO_4_, respectively.

**Fig. 3:**
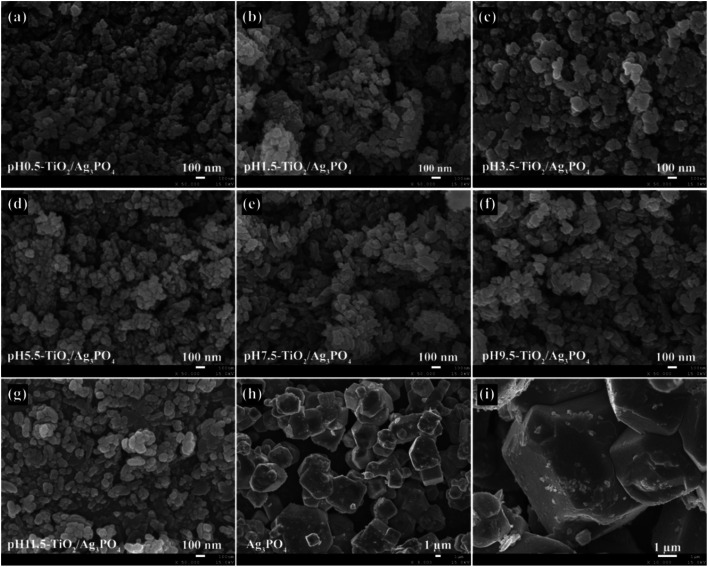
FESEM images of (a) pH 0.5-TiO_2_/Ag_3_PO_4_, (b) pH 1.5-TiO_2_/Ag_3_PO_4_, (c) pH 3.5-TiO_2_/Ag_3_PO_4_, (d) pH 5.5-TiO_2_/Ag_3_PO_4_, (e) pH 7.5-TiO_2_/Ag_3_PO_4_, (f) pH 9.5-TiO_2_/Ag_3_PO_4_, and (g) pH 11.5-TiO_2_/Ag_3_PO_4_ composites specimens, and (h and i) Ag_3_PO_4_ specimens.

Morphology of the TiO_2_/Ag_3_PO_4_ composites and the pure Ag_3_PO_4_ specimens was determined by FESEM. The FESEM images of the TiO_2_/Ag_3_PO_4_ composites that were synthesized under different pH values conditions (pH 0.5–11.5) are shown in Fig. 3(a)–(g). Results show that there were no significant differences in the morphology of the TiO_2_/Ag_3_PO_4_ composites synthesized at pH 0.5–11.5, and all the nanocrystals are severely agglomerated together. When the pH is 0.5, many square rod-shaped anatase nanocrystals with about 70–160 nm in length and 40–50 nm in width, a lot of cuboid-shaped anatase nanocrystals with 25–110 nm in length and 20–60 nm in width, lots of shuttle-like anatase nanocrystals with the size about 60–110 nm in length and 30–50 nm in width and a large number of egg-like anatase nanocrystals with about 30–60 nm in the central axis length and 15–30 nm in the central axis width are observed, as shown in Fig. 3(a). Fig. 3(b) shows the FESEM image of the pH 1.5-TiO_2_/Ag_3_PO_4_ composite, it can seen that some square rod-shaped anatase nanocrystals with about 60–140 nm in length and 30 nm in width, some cuboid-shaped anatase nanocrystals with 35–50 nm in length and 30–40 nm in width, several shuttle-like anatase nanocrystals with about 90–150 nm in length and 45 nm in width, and a large numbers of egg-like anatase nanocrystals with about 20–70 nm in length and 15–40 nm in width are observed. When the pH value rises to 3.5, many cuboid-shaped anatase nanocrystals with 40–80 nm in length and 35–70 nm in width, some shuttle-like anatase nanocrystals with 95–185 nm in length and 40–80 in width, lots of spheroidal anatase nanocrystals with 20–45 nm in diameter, and several diamond-shaped anatase nanocrystals with 50–90 nm in length and 20–60 nm in width are observed, as shown in Fig. 3(c). Fig. 3(d) shows the representation FESEM image of the pH 5.5-TiO_2_/Ag_3_PO_4_ composite prepared by mixed the pH 5.5-TiO_2_ and Ag_3_PO_4_ samples. As shown in Fig. 3(d), egg-like anatase nanocrystals with a size of 30–60 nm and 30–70 nm in length in high yield, some shuttle-like anatase nanocrystals with a size of about 20–50 nm in width and 60–150 nm in length, and cuboid-shaped anatase nanocrystals with a size of 30–70 nm in width and 55–90 nm in length are observed. The FESEM image in Fig. 3(e) shows that the pH 7.5-TiO_2_/Ag_3_PO_4_ composite that has two main morphologies, shuttle-like anatase nanocrystals with 40–200 nm in length and 25–50 nm in width, and cuboid-shaped anatase nanocrystals with 40–95 nm in length and 30–55 nm in width. Fig. 3(f) and (g) show FESEM images of the pH 9.5-TiO_2_/Ag_3_PO_4_ and pH 11.5-TiO_2_/Ag_3_PO_4_ composites, respectively. It can be seen that the prepared composites have similar morphologies, square rod-like (or cuboid-shaped) anatase nanocrystals with a length of about 50–130 nm (or 25–90 nm) and a width of about 30–50 nm (25–70 nm), spheroidal anatase nanocrystals with 20–95 nm (or 20–95 nm) in diameter, and shuttle-like anatase nanocrystals with a length of about 30–210 (or 30–215 nm) nm and a width of about 20–65 (or 20–85 nm) nm for pH 9.5-TiO_2_/Ag_3_PO_4_ (or pH 11.5-TiO_2_/Ag_3_PO_4_) composites. FESEM images of Ag_3_PO_4_ microcrystals are shown in Fig. 3(h) and (i), it can be seen that well-dispersed irregular Ag_3_PO_4_ polyhedrons with about 3–12 μm in length and 2.5–9.0 μm in width (or thickness), and cubic-like particles with the size about 1.5–7.0 μm were obtained. And the surface of the Ag_3_PO_4_ crystals is rough, which is formed by the agglomeration of many nanoparticles with the size about 30–50 nm in diameter (Fig. 3(i)). Based on the above analysis, the Ag_3_PO_4_ crystals were not observed in the TiO_2_/Ag_3_PO_4_ composites, which can be ascribed to the fact that the sizes of Ag_3_PO_4_ crystals were micrometer while the anatase TiO_2_ crystals were nanometer, and TiO_2_ nanocrystals were bound to the surface of Ag_3_PO_4_ microcrystals.

**Fig. 4:**
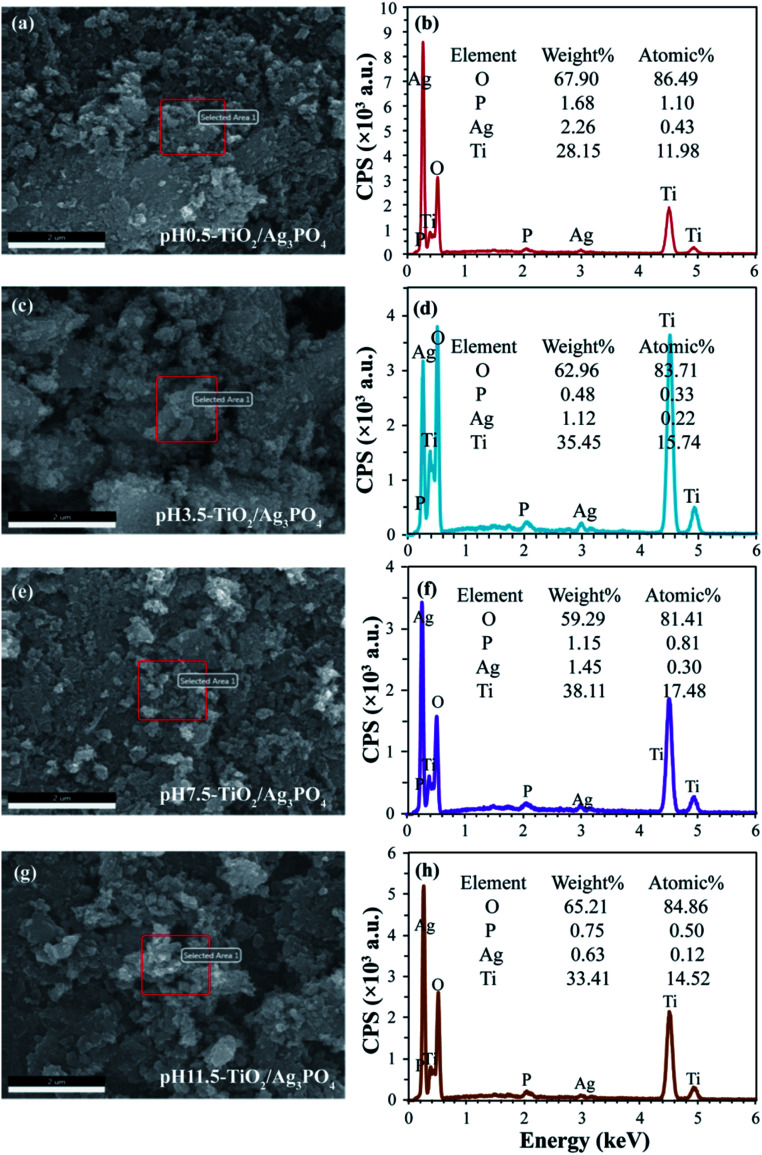
FESEM images and the corresponding EDS with elemental mapping images of (a and b) pH 0.5-TiO_2_/Ag_3_PO_4_, (c and d) pH 3.5-TiO_2_/Ag_3_PO_4_, (e and f) pH 7.5-TiO_2_/Ag_3_PO_4_, and (g and h) pH 11.5-TiO_2_/Ag_3_PO_4_ composites specimens.

The FESEM images and the corresponding elemental distribution maps of TiO_2_/Ag_3_PO_4_ composites were achieved by energy dispersive spectrometer (EDS). As shown in Fig. 4, the appearance of Ag and P elements in EDS further demonstrated successful impregnation of Ag_3_PO_4_. The analysis of the results shows the atomic ratio of Ag to Ti is about 1 : 27.86, 1 : 71.55, 1 : 58.27, and 1 : 121 for pH 0.5-TiO_2_/Ag_3_PO_4_, pH 3.5-TiO_2_/Ag_3_PO_4_, pH 7.5-TiO_2_/Ag_3_PO_4_, and pH 11.5-TiO_2_/Ag_3_PO_4_ composites, respectively.

**Fig. 5:**
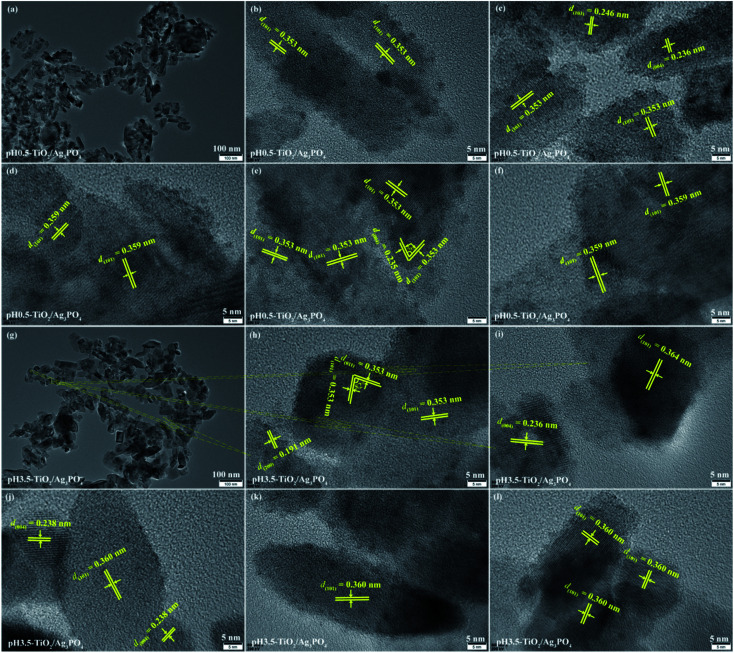
TEM and HRTEM images of (a–f) pH 0.5-TiO_2_/Ag_3_PO_4_ and (g–j) pH 3.5-TiO_2_/Ag_3_PO_4_ composites specimens.

**Fig. 6:**
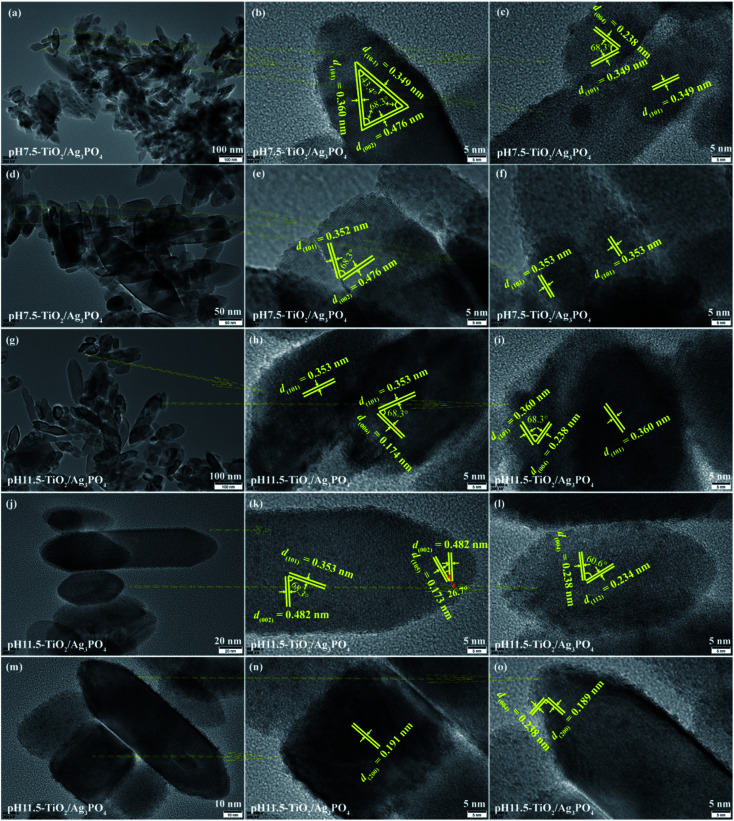
TEM and HRTEM images of (a–f) pH 7.5-TiO_2_/Ag_3_PO_4_ and (g–o) pH 11.5-TiO_2_/Ag_3_PO_4_ composites specimens.

The TEM and HRTEM images further reveal the detailed surface morphology of the obtained TiO_2_/Ag_3_PO_4_ composites products, as shown in Fig. 5 and 6. For pH 0.5-TiO_2_/Ag_3_PO_4_, shuttle-like anatase nanocrystals with the length of about 30–85 nm and the width of about 15–25 nm, and square rod-shaped anatase nanocrystals with the length of about 25–140 nm and the width of about 15–50 nm are observed (Fig. 5(a)), which corresponds to the results of FESEM (Fig. 3(a)). The square rod-shaped nanocrystals with a lattice of 0.353 nm (or 0.359 nm) can be indexed to the (101) planes of the anatase, and the egg-like nanoparticle with a lattice fringes of 0.359 nm also can be indexed to the (101) planes of the anatase (Fig. 5(b)–(f)). The lateral planes of square rod-shaped nanocrystals are parallel to (101) planes, indicating that the exposed facets are {101} facets (Fig. 5(b) and (c)). The lattice fringe has *d*-spacing values of 0.236 and 0.246 nm, corresponding to (004) and (103) planes of anatase TiO_2_, respectively (Fig. 5(c)). The long axis of the shuttle-like anatase nanocrystals is perpendicular to (004) planes, indicating that the exposed facets are {001} facets of the top and bottom planes (Fig. 5(c)). In Fig. 5(e), the lattice fringes of the irregular crystals with lattice spacings of 0.235 and 0.353 nm can be assigned to the (004) and (101) planes of the anatase TiO_2_, respectively. And the angle between the (004) and (101) facets is 68°, implying that the irregular crystals expose {010} facets on its surface. The coexistence of various morphologies of the pH 3.5-TiO_2_/Ag_3_PO_4_ composites was further investigated by TEM and HRTEM, as shown in Fig. 5(j–l). For the cuboid-shaped anatase nanocrystals, the TEM images depict the nanocrystals with 15–50 nm in length and 15–30 nm in width (Fig. 5(g)), and the lattice fringe has *d*-spacing values of 0.353 (or 0.360) and 0.353 nm, corresponding to (101) and (011) planes of anatase TiO_2_, respectively (Fig. 5(h) and (l)). The interior angle between (101) and (011) planes of 82° is in good agreement with the theoretical value, which indicates that the preferentially exposed crystal facets of the cuboid-shaped anatase is perpendicular to [111] crystal zone axis (expressed as [111]-facets). For the shuttle-like anatase nanocrystals, the TEM images depict the nanocrystals with 15–120 nm in length and 10–45 nm in width (Fig. 5(g)), and the lattice fringe has *d*-spacing values of 0.360 nm, corresponding to (101) planes of anatase TiO_2_ (Fig. 5(k)). For the diamond-shaped anatase nanocrystals, the TEM images depict the nanocrystals with 35–85 nm in length and 15–35 nm in width (Fig. 5(g)), and the lattice fringe has *d*-spacing values of 0.191 and 0.360 (or 0.364) nm, corresponding to (200) and (101) planes of anatase TiO_2_, respectively (Fig. 5(h)–(j)). The lateral planes of the diamond-shaped anatase nanocrystals is parallel to (101) planes, indicating that the exposed facets are {101} facets of the lateral planes. For the square rod-shaped anatase nanocrystals, the TEM images depict the nanocrystals with 40–135 nm in length and 20–30 nm in width (Fig. 5(g)), and the lattice fringe has *d*-spacing values of 0.360 nm, corresponding to (101) planes of anatase TiO_2_ (Fig. 5(l)). The top and bottom planes of square rod-shaped anatase nanocrystals are parallel to (101) planes, indicating that the exposed facets are {101} facets.

Fig. 6(a)–(f) shows the TEM and HRTEM analysis results of the pH 7.5-TiO_2_/Ag_3_PO_4_ composite. The size of cuboid-shaped anatase nanocrystals has a size about 30–60 nm in length and 20–35 nm in width, as shown in Fig. 6(a). The size of shuttle-like anatase nanocrystals is about 25–250 nm in length and 20–75 nm in width (Fig. 6(a) and (d)), and the lattice fringe of 0.349, 0.476 and 0.360 nm corresponds to the distance between two adjacent (10-1), (002) and (101) planes of anatase TiO_2_, and the intersection angles between (10-1) and (002), (101) and (002), and (101) and (10-1) planes are 68.3°, 68.3°, and 43.4°, respectively, as shown in Fig. 6(b). The high crystallized shuttle-like TiO_2_ surfaces with the clear lattice fringes of the anatase phase are also observed from Fig. 6(c) and (f). Two set of non-parallel lattice fringes with the *d*-spacing values of 0.349 and 0.238 nm, corresponding to (101) and (004) atomic planes of anatase phase (Fig. 6(c)). The lattice spacing of 0.352 and 0.476 nm of the truncated shuttle-like TiO_2_ anatase TiO_2_, corresponding to the distance between two adjacent (101) or (002) planes, and the intersection angle between (101) and (002) planes is 68.3°, as shown in Fig. 6(e). Based on the above TEM and HRTEM analysis and the Wulff construction model, the shuttle-like anatase TiO_2_ nanocrystals preferentially expose the {010} facets, {101} facets, and {001} facets on the four lateral planes, the eight isosceles trapezoid planes, and the two top/bottom surfaces, respectively, and the directional grown direction is along the [001]-direction. The size of shuttle-like (or cuboid-shaped) anatase nanocrystals is about 50–180 nm (or 25–100 nm) in length and 25–50 nm (or 20–80 nm) in width, as shown in Fig. 6(g, j and m). {010} facets exposed TiO_2_ exhibits a typical shuttle-like morphology with lattice fringes of 0.353 (or 0.360) and 0.174 (or 0.238, 0.482) nm attributed to (101) and (006) (or (004), (002)) crystallographic planes, respectively, and an interfacial angle of 68.3° between the {101} and {001} planes, as shown in Fig. 6(h, i and k). In addition, {010} facets exposed shuttle-like TiO_2_ nanocrystals also has *d*-spacing values of 0.173 (or 0.234, 0.189) and 0.482 (or 0.238, 0.238) nm, corresponding to (105) (or (112), (200)) and (002) (or (004), (004)) crystallographic planes, respectively, and an interfacial angle of 26.7° (or 60.6°, 90°) between the {105} (or (112), (200)) and {112} (or (004), (004)) planes, as shown in Fig. 6(k, l and o). Fig. 6(n) exhibits a typical TEM image of cuboid-shaped anatase nanocrystals, the fringe spacing of 0.191 nm corresponding to the (200) planes of anatase TiO_2_, indicating that the exposed crystal facets of the top/bottom of the nanocrystals are {100} facets.

**Fig. 7:**
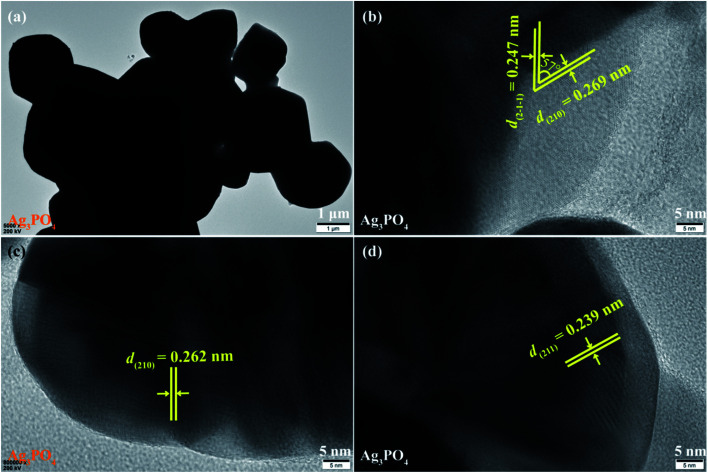
(a) TEM and (b–d) HRTEM images of Ag_3_PO_4_ specimens.

The morphology and microstructure of the Ag_3_PO_4_ crystals were further analyzed by TEM and HRTEM images, as shown in Fig. 7. As can be seen in Fig. 7(a), the obtained Ag_3_PO_4_ crystals contains some irregular polyhedrons with the lengths of 1.0–3.7 μm and a cubic-like crystals with the lengths of about 1.75 μm and the widths of about 1.45 μm, respectively, which is in agreement with the results observed by the SEM images (Fig. 3(h) and (i)). The lattice fringes of 0.269 (or 0.262) and 0.247 (or 0.239) nm match well with the (210) and (2-1-1) (or (211)) planes of irregular polyhedral Ag_3_PO_4_ crystals, respectively (Fig. 7(b)–(d)). And the angle between the (210) and (2-1) facets of 57° agrees well with the theoretical value 56.8°, according to calculated result from the lattice constants of Ag_3_PO_4_ (cubic, space group *P*43̄*n*, JCPDS 06-0505, and *a* = 6.013 Å). Based on the above TEM and HRTEM analysis, the Ag_3_PO_4_ specimens in the TiO_2_/Ag_3_PO_4_ composites were not observed, which can be attributed to the deposition of nanoscale anatase TiO_2_ crystals on the microsized Ag_3_PO_4_ crystals *via* an *in situ* precipitation process.

### 3.2 Growth mechanism of the TiO_2_/Ag_3_PO_4_ composites

According to the results of the XRD, SEM and HR-TEM observation, the possible growth mechanism for the formation of TiO_2_/Ag_3_PO_4_ hybrids can be expressed as follows. Firstly, the TMA^+^-intercalated H_2_Ti_4_O_9_/H_2_Ti_2_O_5_ compounds (TMA^+^-H_2_Ti_4_O_9_/H_2_Ti_2_O_5_) were exfoliated into nanosheets solutions under stirring conditions.

1 TMA^+^-H_2_Ti_4_O_9_ + H_2_O → TMA^+^ + [Ti_4_O_9_]^2−^ + H_3_O^+^

2 TMA^+^-H_2_Ti_2_O_5_ + H_2_O → TMA^+^ + [Ti_2_O_5_]^2−^ + H_3_O^+^

The positive ions of TMA^+^ and H_3_O^+^ located on surface of [Ti_4_O_9_]^2−^/[Ti_2_O_5_]^2−^ nanosheets to balance the negative charge of [Ti_4_O_9_]^2−^/[Ti_2_O_5_]^2−^ so that the nanosheets remain electrically neutral. Then, the nanosheets solutions containing of [Ti_4_O_9_]^2−^/[Ti_2_O_5_]^2−^ compounds (pH = 0.5–11.5) were transformed to anatase TiO_2_ nanocrystals under hydrothermal conditions by the following reaction.

3 [Ti_4_O_9_]^2−^ + 2H^+^ → 4TiO_2_ + H_2_O

4 [Ti_2_O_5_]^2−^ + 2H^+^ → 2TiO_2_ + H_2_O

5 [Ti_4_O_9_]^2−^ + H_2_O → 4TiO_2_ + 2OH^−^

6 [Ti_2_O_5_]^2−^ + H_2_O → 2TiO_2_ + 2OH^−^

Acidic condition is beneficial for reactions (3) and (4), neutral and basic conditions are favorable for reactions (5) and (6). In this process, the [Ti_4_O_9_]^2−^/[Ti_2_O_5_]^2−^ nanosheets were transformed firstly to nanosheet-like anatase TiO_2_ crystals by an *in situ* topotactic dehydration reaction.^37^ Then the nanosheet-like anatase TiO_2_ crystals were split into anatase TiO_2_ nanocrystals with various morphologies and different exposed facets by dissolution–recrystallization process along their different planes.

The micro-sized Ag_3_PO_4_ crystals were synthesized by using an ion-exchange method, using AgNO_3_ and NH_4_H_2_PO_4_ (3Ag^+^ + H_2_PO_4_^−^ = Ag_3_PO_4_↓ + 2H^+^). The anatase TiO_2_ nanocrystals with various morphologies and different exposed facets and Ag_3_PO_4_ precipitate were well dispersed into deionized water under stirring to form suspension solution. The micro-sized Ag_3_PO_4_ polyhedrons with larger particle surface, which could absorb more nano-sized anatase TiO_2_ nanocrystals onto their surfaces *via* an *in situ* precipitation process to form the heterostructured TiO_2_/Ag_3_PO_4_ composites.

### 3.3 UV-vis adsorption spectra of Ag_3_PO_4_, TiO_2_/Ag_3_PO_4_ and TiO_2_

**Fig. 8:**
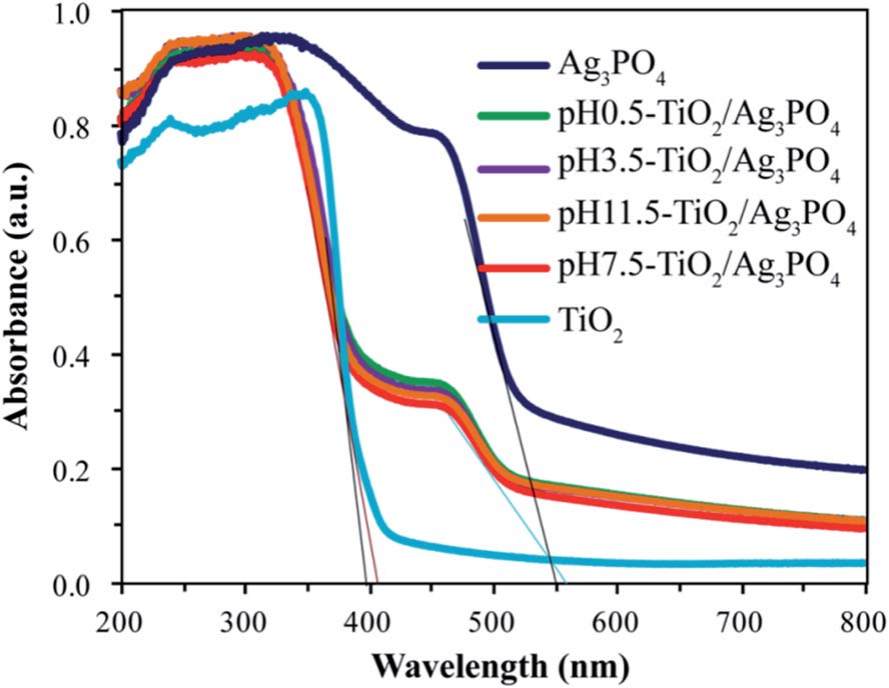
UV-Vis NIR Spectra of pure TiO_2_, pure Ag_3_PO_4_, pH 0.5-TiO_2_/Ag_3_PO_4_, pH 3.5-TiO_2_/Ag_3_PO_4_, pH 7.5-TiO_2_/Ag_3_PO_4_, and pH 11.5-TiO_2_/Ag_3_PO_4_ composites.

The UV-visible absorption spectrum was applied to examine the optical properties of pure Ag_3_PO_4_, TiO_2_ and TiO_2_/Ag_3_PO_4_ composites. As observed in Fig. 8, the UV-Vis NIR spectrum of pure TiO_2_ sample only exhibits the fundamental absorption band edge (395 nm) in the UV light region, and the absorption band edge almost no more exists in the visible wavelength range. The pure Ag_3_PO_4_ sample shows strong adsorption with absorption band edge at around 500 nm, which is equivalent to the band gap energy of 2.45 eV, in an good agreement with the results reported previously.^38^ However, for the prepared TiO_2_/Ag_3_PO_4_ composites at different values of pH, except for adsorption band edge (less than 408 nm) in the UV light region, a feature band edge (510 nm) of pure Ag_3_PO_4_ appears in the visible light range based on the UV-Vis NIR spectrum. The absorption edges of TiO_2_/Ag_3_PO_4_ composites are shifted slightly toward higher wavelength relative to pure Ag_3_PO_4_, indicating TiO_2_ in the composites is coupled to Ag_3_PO_4_. The above analysis show that the as-prepared TiO_2_/Ag_3_PO_4_ composites can be used for visible light photocatalytic reactions.

### 3.4 Photocatalytic activities for the degradation of rhodamine B solutions

**Fig. 9:**
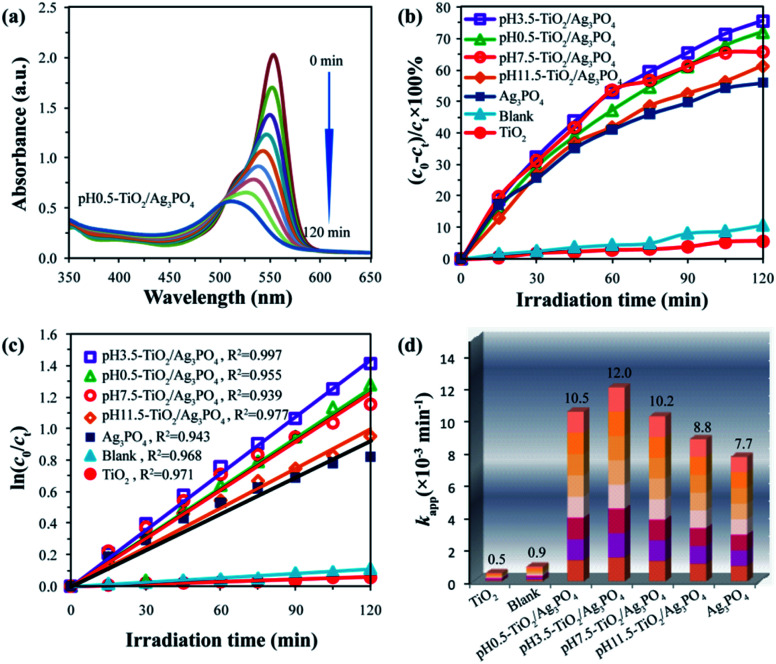
(a) Temporal evolution of rhodamine B (RhB) over pH 0.5-TiO_2_/Ag_3_PO_4_ composite, (b) photocatalytic degradation of RhB solution over blank, TiO_2_, Ag_3_PO_4_, and TiO_2_/Ag_3_PO_4_ composites under Xe light irradiation with a 400 nm cutoff filter, (c) the kinetic fit of the RhB degradation by blank, TiO_2_, Ag_3_PO_4_, and TiO_2_/Ag_3_PO_4_ composites, and (d) the corresponding apparent rate constants.

Recently, different types of photocatalysts, such as Mn-doped ZrO_2_,^39^ carbon quantum dots,^40^ MOFs,^11^ BaTiO_3_,^41^ were used to degrade the organic pollutants. In this study, the photocatalytic activities of the TiO_2_/Ag_3_PO_4_ composites were evaluated by degradation of the carcinogenic textile dye rhodamine B (RhB, adsorption band: 554 nm). The degradation efficiency of all the specimens is expressed as (*c*_0_ − *c*_*t*_)/*c*_0_ × 100%, where *c*_0_ and *c*_*t*_ represent the initial and residual concentration of the RhB, respectively. Prior to illumination, the suspensions were magnetically stirred in the dark for 2 h to make the RhB dyes reach achieve adsorption–desorption equilibrium on the surface of TiO_2_/Ag_3_PO_4_ composites.^42^ The adsorption values (mol(RhB) g(TiO_2_/Ag_3_PO_4_)^−1^) of RhB on the surface of TiO_2_/Ag_3_PO_4_ composites were 4.0 × 10^−6^, 7.0 × 10^−6^, 5.5 × 10^−6^, and 4.5 × 10^−6^ mol g^−1^ for pH 0.5-TiO_2_/Ag_3_PO_4_, pH 3.5-TiO_2_/Ag_3_PO_4_, pH 7.5-TiO_2_/Ag_3_PO_4_, and pH 11.5-TiO_2_/Ag_3_PO_4_ samples, respectively. These results indicated that the enhancement order of adsorption binding of the RhB to the TiO_2_/Ag_3_PO_4_ was pH 0.5-TiO_2_/Ag_3_PO_4_ < pH 11.5-TiO_2_/Ag_3_PO_4_ < pH 7.5-TiO_2_/Ag_3_PO_4_ < pH 3.5-TiO_2_/Ag_3_PO_4_, and that the strong anchoring of the RhB onto the surface of pH 3.5-TiO_2_/Ag_3_PO_4_ could improve the photocatalytic activity. The commercial TiO_2_ powder (∼70.9% anatase and ∼29.1% rutile) and Ag_3_PO_4_ powder were used as the photocatalytic references. Fig. 9(a) shows the variation of the absorption of rhodamine B (RhB) in the presence of pH 0.5-TiO_2_/Ag_3_PO_4_ composite under the Xe light irradiation for 120 min. The peak position at 554 nm gradually moved towards the short-wavelength direction (*i.e.*, hypsochromic shift) and the intensity gradually decreased, indicating the partial *N*-de-ethylation and the destruction of structure of the polycyclic aromatic hydrocarbon by the gradual decolorization of the RhB solution.^43^

**Fig. 10:**
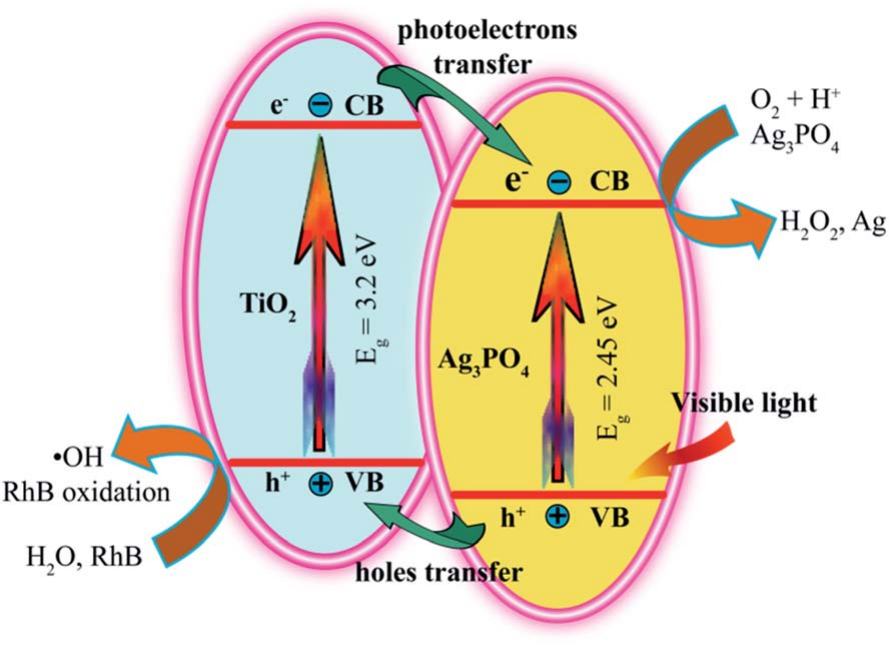
Possible photocatalytic mechanism for the photodegradation of RhB over the TiO_2_/Ag_3_PO_4_ composites under visible light irradiation.

After exposure to visible light for 120 min, the degradation of RhB was as follows: pH 3.5-TiO_2_/Ag_3_PO_4_ (75.6%) > pH 0.5-TiO_2_/Ag_3_PO_4_ (72.2%) > pH 7.5-TiO_2_/Ag_3_PO_4_ (65.8%) > pH 11.5-TiO_2_/Ag_3_PO_4_ (61.3%) > Ag_3_PO_4_ (56.0%) > blank (10.8%) > the commercial TiO_2_ (5.8%), as shown in Fig. 9(b). Obviously, the as-prepared TiO_2_/Ag_3_PO_4_ composites exhibit enhanced photocatalytic performance for the degradation of RhB compared to the commercial TiO_2_ powder and Ag_3_PO_4_ powder. The enhanced photocatalytic performance can be attributed to the TiO_2_/Ag_3_PO_4_ heterostructures, which can absorb more visible light and inhibit the recombination of photoelectrons and holes.^34^Fig. 10 shows a possible photocatalytic mechanism for the photodegradation of RhB over the TiO_2_/Ag_3_PO_4_ heterostructures under visible light irradiation. The valence band (VB) potential (+2.90 eV *vs.* NHE) and conduction band (CB) potential (+0.45 eV *vs.* NHE) of Ag_3_PO_4_ are more positive than those of TiO_2_ (VB potential: +2.70 eV, and CB potential: −0.30 eV), which imply that the photon generated electrons (e^−^) of TiO_2_ nanocrystal will be quickly transferred to the CB of Ag_3_PO_4_ crystal, whereas the photon generated holes (h^+^) of Ag_3_PO_4_ crystal will be migrated to the VB of TiO_2_ nanocrystal under visible light irradiation.^30,44^ The separation of the e^−^ (in Ag_3_PO_4_ crystal) and h^+^ (in TiO_2_ nanocrystal) inhibits the charge recombination, which leads to the improvement of the photocatalytic activity of TiO_2_/Ag_3_PO_4_ composites.^32^ The h^+^ and e^−^ have oxidation and reduction, respectively. Under visible light irradiation, the h^+^ in the VB of TiO_2_ nanocrystal can directly oxidize the organic dye RhB and the water molecules adsorbed to the surface of TiO_2_ photocatalyst to form RhB oxidation and ˙OH radicals, respectively.^3^ At the same time, the e^−^ in the CB of Ag_3_PO_4_ crystal can directly reduce the oxygen molecules adsorbed to the surface of Ag_3_PO_4_ photocatalyst to form strong oxidizing capacity of hydrogen peroxide (H_2_O_2_) to oxidize and degradation RhB. Moreover, Ag_3_PO_4_ is reduced to Ag by e^−^ in the photocatalytic process. The 10 mg L^−1^ RhB solution (10 ppm) was not completely degraded due to the addition of more RhB solution (150 mL) and fewer catalysts (75 mg), and the liquid level of RhB solution was far away from the light source (25 cm). However, TiO_2_ exhibited very low photocatalytic activity for the photodegradation of RhB, only 5.8% degradation efficiency, even lower than 10.8% for the blank without any photocatalysts under the Xe light irradiation for 120 min, implying that the TiO_2_ actually had no any photocatalytic activity. Based on the discussion results of TiO_2_ and the blank, it is reasonable that the presence of photocatalyst has a shielding effect on the degradation of RhB under the Xe light irradiation.^45^

Since the process of photodegradation of RhB solution followed the pseudo-first-order reaction kinetics model, the fitted pseudo-first-order reaction plots, the correlation coefficient and the corresponding apparent rate constant (*k*_app_) are shown in Fig. 9(c) and (d), respectively. The correlation coefficients (*R*^2^) are 0.968, 0.971, 0.943, 0.955, 0.997, 0.939, and 0.977 for the blank, TiO_2_, Ag_3_PO_4_, pH 0.5-TiO_2_/Ag_3_PO_4_, pH 3.5-TiO_2_/Ag_3_PO_4_, pH 7.5-TiO_2_/Ag_3_PO_4_, and pH 11.5-TiO_2_/Ag_3_PO_4_, respectively. The pH 3.5-TiO_2_/Ag_3_PO_4_ composite exhibited the highest *k*_app_ value (12.0 × 10^−3^ min^−1^), which was approximately 24.0, 13.3, 1.6, 1.4, 1.2, and 1.1 times larger than those of the commercial TiO_2_ (0.5 × 10^−3^ min^−1^), blank (0.9 × 10^−3^ min^−1^), Ag_3_PO_4_ (7.7 × 10^−3^ min^−1^), pH 11.5-TiO_2_/Ag_3_PO_4_ (8.8 × 10^−3^ min^−1^), pH 7.5-TiO_2_/Ag_3_PO_4_ (10.2 × 10^−3^ min^−1^), and pH 0.5-TiO_2_/Ag_3_PO_4_ (10.5 × 10^−3^ min^−1^) samples, respectively. The pH 3.5-TiO_2_/Ag_3_PO_4_ composite had the highest *k*_app_ value, indicating that the pH 3.5-TiO_2_/Ag_3_PO_4_ composite had the highest photocatalytic activity.

**Fig. 11:**
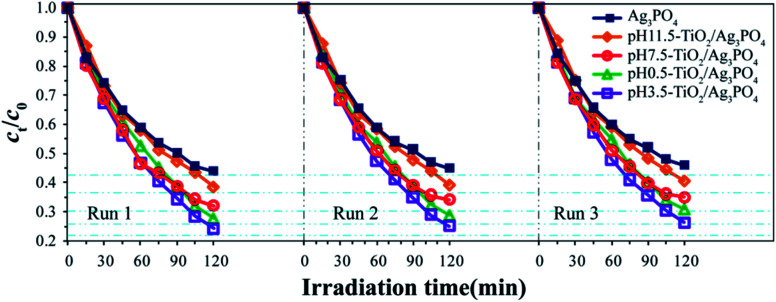
Recycling studies of the pure Ag_3_PO_4_, pH 0.5-TiO_2_/Ag_3_PO_4_, pH 3.5-TiO_2_/Ag_3_PO_4_, pH 7.5-TiO_2_/Ag_3_PO_4_, and pH 11.5-TiO_2_/Ag_3_PO_4_ photocatalysts for the photocatalytic degradation of RhB solution.

The stability and recyclability of photocatalyst is one of the important parameters for its practical applications. Herein, the stability and recyclability of the pure Ag_3_PO_4_ and TiO_2_/Ag_3_PO_4_ composites were evaluated by examining their recyclability in the photodegradation of RhB. Fig. 11 exhibited the repetitive photocatalytic degradation of RhB solution (10 mg L^−1^, 150 mL) during three sequential runs under identical conditions. After each run, TiO_2_/Ag_3_PO_4_ and Ag_3_PO_4_ photocatalysts were collected by centrifugation and washed with deionized water for several times and the fresh RhB solutions with the same concentration (10 mg L^−1^) were used for next run. The photocatalytic efficiency of the TiO_2_/Ag_3_PO_4_ and Ag_3_PO_4_ photocatalysts remained almost unchanged after three successive experimental runs, indicating that the synthesized photocatalysts had remarkable stability.

As is well-known, the photocatalytic activity is not only related to the heterostructure of TiO_2_ nanocrystals, but also influenced by other factors, such as crystalline phase, crystalline size, crystallinity, specific surface area, exposed facets, and so on.^46−48^ For the synthesized TiO_2_/Ag_3_PO_4_ composites, the crystalline form (anatase) and proportion (*w*(TiO_2_) : *w*(Ag_3_PO_4_) = 4 : 1) of TiO_2_ are the same, indicating that the influence of TiO_2_ crystal form and proportion on photocatalytic activity in TiO_2_/Ag_3_PO_4_ composites is negligible. In the TiO_2_/Ag_3_PO_4_ composites, the average crystalline sizes of pH 0.5-TiO_2_/Ag_3_PO_4_, pH 3.5-TiO_2_/Ag_3_PO_4_, pH 7.5-TiO_2_/Ag_3_PO_4_, and pH 11.5-TiO_2_/Ag_3_PO_4_ were 58.5, 68.5, 87.5 and 80.0 nm, respectively, by measuring 200 particles in the FESEM images with Particle Size Distribution Calculation Software (Fudan University, China). And the specific surface areas were 32.6, 27.8, 21.8, 24.4 m^2^ g^−1^ for pH 0.5-TiO_2_/Ag_3_PO_4_, pH 3.5-TiO_2_/Ag_3_PO_4_, pH 7.5-TiO_2_/Ag_3_PO_4_, and pH 11.5-TiO_2_/Ag_3_PO_4_, respectively. It is known that smaller crystal size and larger specific surface (favorable for the RhB adsorption) contribute to the enhancement of photocatalytic activity in the photochemical reaction, which is attributed to its strong oxidation-reduction capability and more active sites.^49,50^ However, the pH 3.5-TiO_2_/Ag_3_PO_4_ composite displayed the highest photocatalytic activity, although the crystal size (68.5 nm) is much bigger than that (58.5 nm) of the pH 0.5-TiO_2_/Ag_3_PO_4_ composite, and the specific surface area (27.8 m^2^ g^−1^) slightly smaller than that (32.6 m^2^ g^−1^) of the pH 0.5-TiO_2_/Ag_3_PO_4_ composite, indicating that it is also very significant to establish a balance between crystal size and specific surface area to improve the photocatalytic performance. On the other hand, the crystallinity of the pH 0.5-TiO_2_/Ag_3_PO_4_ composite is better than that of pH 3.5-TiO_2_/Ag_3_PO_4_ composite, which inhibits the recombination of photogenerated charge carriers (photogenerated electrons and holes), resulting in a relative good photoactivity.^15^

The surface energies of {101}, {010} (or {100}), {001}, {110} and [111]-facets of anatase TiO_2_ are 0.44, 0.53, 0.90, 1.09, and 1.61 J m^−2^, respectively.^21,51^ In generally, in the photocatalytic reaction, the crystal surface with high-energy crystal facets usually exhibits high photocatalytic activity. Based on the discussion above, the anatase TiO_2_ nanocrystals preferentially co-exposed the {101}/{001}/{010} facets, {101}/{001}/[111]-facets, {101}/{010} facets, {101}/{010} (or {100}) facets on their surfaces in the pH 0.5-TiO_2_/Ag_3_PO_4_, pH 3.5-TiO_2_/Ag_3_PO_4_, pH 7.5-TiO_2_/Ag_3_PO_4_, pH 11.5-TiO_2_/Ag_3_PO_4_ composites, respectively. Hence, the improvement of photocatalytic activity of the pH 3.5-TiO_2_/Ag_3_PO_4_ can also be attributed to the coexistence of high-energy {001} and [111]-facets. According to the discussion above, the pH 0.5-TiO_2_/Ag_3_PO_4_ composite possesses a relative small crystal size, large specific surface area, good crystallinity, and co-exposed high-energy {001} and [111]-facets, the synergistic effects resulting in the highest photocatalytic activity of the pH 3.5-TiO_2_/Ag_3_PO_4_ composite.

## 4. Conclusions

In summary, TiO_2_/Ag_3_PO_4_ composites composed of anatase TiO_2_ nanocrystals with co-exposed {101}, {010}/{100}, {001} and [111]-facets and Ag_3_PO_4_ microcrystals with irregular polyhedrons and cubic-like crystals were successfully synthesized by combining hydrothermal and ion-exchange methods. The Ag_3_PO_4_ microcrystals were used as a substrate to load the anatase TiO_2_ nanocrystals on their surface and to form TiO_2_/Ag_3_PO_4_ heterostructures. To investigate the photocatalytic performance, the carcinogenic RhB solution was selected as model pollutant because it widely used in the textile industry. Compared with the commercial TiO_2_ and the pure Ag_3_PO_4_ microcrystals, the heterostructured TiO_2_/Ag_3_PO_4_ composites exhibited excellent photocatalytic activity for the degradation of rhodamine B under visible light irradiation, which can be attributed to the separation of the e^−^ (in Ag_3_PO_4_ crystal) and h^+^ (in TiO_2_ nanocrystal) inhibits the charge recombination. For the as-prepared TiO_2_/Ag_3_PO_4_ composites, the pH 3.5-TiO_2_/Ag_3_PO_4_ composite exhibited the highest photocatalytic activity which mainly attributed to the synergistic effects of its relative small crystal size, large specific surface area, good crystallinity, and co-exposed high-energy {001} and [111]-facets. Moreover, this study provides new way for the preparation of TiO_2_/Ag_3_PO_4_ composite semiconductor photocatalysts with high energy crystal surfaces. However, although the as-prepared TiO_2_/Ag_3_PO_4_ composites exhibited good stability, the photocatalytic performance needs to be further improved for their practical application.

## Conflicts of interest

The authors declare no conflict of interest.

## Supplementary Material
